# Comparing Email Versus Text Messaging as Delivery Platforms for Supporting Patients With Major Depressive Disorder: Noninferiority Randomized Controlled Trial

**DOI:** 10.2196/59003

**Published:** 2024-09-09

**Authors:** Medard K Adu, Oghenekome Eboreime, Reham Shalaby, Ejemai Eboreime, Belinda Agyapong, Raquel da Luz Dias, Adegboyega O Sapara, Vincent I O Agyapong

**Affiliations:** 1 Department of Psychiatry Faculty of Medicine Dalhousie University Halifax, NS Canada; 2 Reproductive Care Program, Healthy Population & Provincial Initiatives IWK Halifax, NS Canada; 3 Department of Psychiatry Faculty of Medicine and Dentistry University of Alberta Edmonton, AB Canada

**Keywords:** major depressive disorder, Text4Support, SMS text messaging, email messaging, digital health, mental health, mobile phone, depressive disorder, health communication, global health, treatments, patient, text messaging-based, cognitive behavioral therapy, communication, effectiveness, mental health support, digital intervention, digital interventions, mental health care, well-being, depression symptoms

## Abstract

**Background:**

The prevalence of major depressive disorder (MDD) poses significant global health challenges, with available treatments often insufficient in achieving remission for many patients. Digital health technologies, such as SMS text messaging–based cognitive behavioral therapy, offer accessible alternatives but may not reach all individuals. Email communication presents a secure avenue for health communication, yet its effectiveness compared to SMS text messaging in providing mental health support for patients with MDD remains uncertain.

**Objective:**

This study aims to compare the efficacy of email versus SMS text messaging as delivery platforms for supporting patients with MDD, addressing a critical gap in understanding optimal digital interventions for mental health care.

**Methods:**

A randomized noninferiority pilot trial was conducted, comparing outcomes for patients receiving 6-week daily supportive messages via email with those receiving messages via SMS text message. This duration corresponds to a minimum of 180 days of message delivery. The supportive messages maintained consistent length and structure across both delivery methods. Participants (N=66) were recruited from the Access 24/7 clinic in Edmonton, Alberta, among those who were diagnosed with MDD. The outcomes were measured at baseline and 6 months after enrollment using the Patient Health Questionnaire-9 (PHQ-9), Generalized Anxiety Disorder-7 (GAD-7), and the World Health Organization Well-Being Index (WHO-5).

**Results:**

Most of the participants were females (n=43, 65%), aged between 26 and 40 years (n=34, 55%), had high school education (n=35, 58%), employed (n=33, 50%), and single (n=24, 36%). Again, most participants had had no history of any major physical illness (n=56, 85%) and (n=61, 92%) responded “No” to having a history of admission for treatment of mood disorders. There was no statistically significant difference in the mean changes in PHQ-9, GAD-7, and WHO-5 scores between the email and SMS text messaging groups (mean difference, 95% CI: –1.90, 95% CI –6.53 to 2.74; 5.78, 95% CI –1.94 to 13.50; and 11.85, 95% CI –3.81 to 27.51), respectively. Both supportive modalities showed potential in reducing depressive symptoms and improving quality of life*.*

**Conclusions:**

The study’s findings suggest that both email and SMS text messaging interventions have equivalent effectiveness in reducing depression symptoms among individuals with MDD. As digital technology continues to evolve, harnessing the power of multiple digital platforms for mental health interventions can significantly contribute to bridging the existing treatment gaps and improving the overall well-being of individuals with depressive conditions. Further research is needed with a larger sample size to confirm and expand upon these findings.

**Trial Registration:**

ClinicalTrials.gov NCT04638231; https://www.ncbi.nlm.nih.gov/pmc/articles/PMC8552095/

## Introduction

### Overview

Major depressive disorder (MDD) is one of the most prevailing mental health conditions, impacting over 300 million individuals worldwide [1]. The lifetime prevalence of MDD is purged at 15% [2], presenting a significant socioeconomic burden and an estimated annual cost of approximately US $236 billion in the United States and €91 billion (equivalent to US $96 billion) in Europe [3,4]. According to data, the lifetime prevalence of MDD in Canada is at 9.9% [5], with an associated yearly economic cost of around US $12 billion [6]. Data suggest that, despite the robust first-line treatment available for individuals diagnosed with MDD, about 50% of these patients do not attain remission, with two-thirds requiring additional treatment attempts before achieving remission [7,8]. Therefore, MDD is a significant contributor to the global burden of disease, and it is projected by the World Health Organization to emerge as the leading cause of global disability by 2030 [9].

Psychopharmacological and nonpharmacological agents, such as psychosocial interventions, are some of the first-line treatment strategies for the management of individuals diagnosed with MDD. Nevertheless, access to these psychotherapies is restricted due to limitations in human resource capacity, which leaves the majority of individuals with MDD going untreated [10,11].

Geographical location further limits access as most of these psychosocial treatment modalities are commonly found within cities and towns compared to rural areas [12,13]. Even within the urban areas, these services are mostly available during regular working daytime and business hours. Consequently, caregivers frequently find themselves grappling with workloads that are 2 or more times the recommended amount, significantly reducing the availability of appointments [14,15]. The problem is further compounded by prolonged patient wait times for counseling services and the related stigma with seeking these services.

Evidence in the literature underscores the effectiveness and cost efficiency of emerging health service technologies such as the tele mental health with its potential to enhance improvement in access to mental health care and, thereby, aiding in the closure of the prevailing treatment gaps [16,17]. Some effective means of providing health services include email messaging, smartphone apps, and SMS text messaging [17,18]. Care providers must adopt these evolving trends in the digital space for care delivery, especially in mental health care.

According to a data report in 2019, approximately 97% of the global population lived in communities with access to mobile cellular signals [19]. This presupposes the widespread accessibility of mobile networks. The surge in mobile phone usage has significantly created a high possibility for advancing health promotion and care delivery leading to an exponential growth in mobile health (mHealth) in recent years [20]. A global survey conducted in 2015 on the usage of internet services among Western countries highlighted Canada’s high internet usage around 90%. The data also demonstrated that about 67% of Canadians owned smartphones [21].

Though some evidence in the literature supports the clinical and cost-effectiveness of internet-based mental health services [22], most health care givers have, until recently, been slow to adopt this clinical treatment approach [23,24]. Technology-powered cognitive behavioral therapy (CBT) interventions such as supportive SMS text messages are recognized as an innovative, accessible, and cost-effective means for the delivery of psychological care to individuals and the general public with mental health concerns [25]. Data suggest an estimated 99% of SMS text messages received are opened, and about 90% of these messages are read within 3 minutes of their reception [26], thus offering an essential, accessible means of delivering quality, cost-effective psychological care with the potential of closing the huge treatment gap that exists for patients with depressive conditions [27].

Text4Support is an SMS text message–based form of CBT that allows individuals diagnosed with MDD to receive daily supportive SMS text messages that seek to correct or alter negative thought patterns through positive reinforcement. Text4Support is part of the ResilienceNHope [28] suite of supportive SMS text messages offered by the Global Psychological eHealth Foundation [29]. The program provides users with daily supportive SMS text messages created by a team of CBT therapists and mental health experts in collaboration with mental health service users. Text4Support is designed to improve the mood of individuals diagnosed with MDD using positive reinforcement to correct distorted or negative thought patterns.

The effectiveness of Text4Support is backed by data generated from randomized control trials conducted in persons diagnosed with major depressive illness and who received twice daily supportive SMS text messages. The studies revealed that there was a significant reduction in the participants’ depressive symptoms [30,31]. After 3 months of daily supportive SMS text messages to participants, the initial study conducted in Ireland recorded a mean difference of –7.9 (95% CI –13.06 to –2.76; Cohen *d*=0.85) in Becks Depression Inventory (BDI)-II scores between the intervention and the control group [30]. A subsequent Irish study with a larger sample size recorded similar findings. In the study conducted on a Canadian sample, after 3 months of exposure to daily supportive SMS text messages to the participants, the results demonstrated a significant difference in the means of the intervention and control groups as recorded on the BDI scale 20.8 (SD 11.7) versus 24.9 (SD 11.5), respectively, *F*_1, 60_=4.83; *P*=.03; ηp^2^=0.07, with the corresponding effect size (Cohen *d*) of 0.67 [31].

While the popularity and patronage of supportive SMS text messaging interventions are generally high, anecdotally, some individuals were faced with challenges preventing them from subscribing to some of the supportive SMS text messaging interventions, such as the Text4Mood [27] and Text4Hope [32] programs due to the lack of personal and active cell phones. As part of the satisfaction survey conducted for the Text4Hope intervention, about 64% of the participants registered a preference to receive health care support via email during crises [32].

Email is widely used as a means of communication on a global scale and has become integral to the daily lives of many individuals, both personally and for professional concerns. Despite its prevalence, its adoption within the health care sector has been comparatively lower than in other industries. Emails, widely used in web-based interventions, offer asynchronous, low-cost support [33,34]. However, variations in their usage make comparisons challenging, leading to mixed clinical results [35-37]. Automated emails require minimal monitoring, while individually crafted ones demand more effort [34].

As e-mental health apps gain prominence, emails have become a secure avenue of health communication, especially in the interactions between patients and their care providers [38]. The unique attributes of email messages, for example, are the flexibility of the length of messages, fast message delivery, and asynchronous communication, which contribute to its distinctiveness [39]. Furthermore, enhanced communication via email allows patients to ask questions they may have forgotten during previous encounters, while physicians can provide detailed information and offer further assistance if needed. Electronic correspondence enhances the perception of provider availability and enables real-time reporting of results, reducing the time to inform patients. Additionally, transmitting treatment instructions electronically can decrease patient visits, mitigate disease transmission risks, improve patient flow, and reduce copays without sacrificing care quality in areas with prevalent communicable diseases such as COVID-19 [40]. Currently, there is limited understanding of how patients use email for health care interactions, but such insights are crucial for evaluating the effectiveness of proposed policies and the impact of email communication. Key factors influencing the adoption of email communication between patients and health care professionals include patient health status, disparities in digital access, and the potential effects on health care service use. Compared to immediate interactions through video, telephone, or in-person meetings, email provides the opportunity to send a specific question that can be answered later, potentially hours or days after. Email communication, whether through an enterprise email platform such as Outlook (Microsoft Corporation) or a patient portal, offers various uses and benefits [40]. Given the limited data regarding the use and effectiveness of email for health care interactions, this study seeks to determine whether delivering supportive messages via email can be as effective as SMS text messaging in providing mental health support for patients diagnosed with MDD.

### Study Aims and Objectives

This study aims to evaluate and compare the effectiveness of SMS text messaging and email messaging strategies for delivering CBT-based supportive messages to patients with MDD. The specific objectives were to compare the mean differences in the Patient Health Questionnaire-9 (PHQ-9) scale, the Generalized Anxiety Disorder-7 (GAD-7) scale, and the World Health Organization Well-Being Index (WHO-5) scores from baseline to 6 months for the participants receiving daily supportive email messages with those receiving daily supportive SMS text messages.

### Hypothesis

Given that the same supportive messages were delivered through both the email and SMS text messaging programs, our research hypothesis was that the email messaging program would not be inferior to the SMS text messaging program. That is, there would be no significant differences in the mean change scores from baseline to 6 weeks in participants based on the mode of delivery of the messages.

## Methods

### Study Design, Participants, and Settings

This study is a randomized noninferiority pilot trial testing the comparative effectiveness of SMS text message versus email daily supportive SMS text messages for mitigating depression symptoms in patients diagnosed with MDD. Participants were recruited from the Alberta Health Services Addiction and Mental Health Access 24/7 clinic located in Edmonton, Alberta, Canada, a zone-wide centralized intake clinic for addiction and mental health. Patients with mental health concerns can self-present or be referred by a primary care provider to the Access 24/7 clinic for assessments and connection to other community mental health clinics for follow-up or to other community supports as necessary. Patients who had been assessed by a psychiatrist at the Access 24/7 clinic and diagnosed with an MDD according to the *DSM-5* (*Diagnostic and Statistical Manual for Mental Disorders* [Fifth Edition]) using a structured clinical interview were offered an information leaflet about the study and, if agreeable, were invited to participate in the study by signing a consent form.

### Inclusion Criteria

Participants were included if they were (1) aged 18 years and older who could provide informed consent, (2) patients who had been assessed using structured clinical interviews for *DSM-5* and diagnosed with an MDD, (3) patients who had a cell phone with an active line and a functional email address could access both email messages and SMS text messages, and (4) patients who provided informed consent.

Based on the clinical assessment conducted by the psychiatrists at the Access 24/7 clinic, patients were ineligible if they had active psychotic disorders or mental health conditions other than MDD or resided outside of regular cell phone and internet connection areas. Also, patients were ineligible if they were previously or currently subscribed to a supportive SMS text messaging program.

### Sample Size Calculation

The effect sizes (Cohen *d*) achieved in 2 previous randomized trials (n<80 for each trial), which compared daily supportive SMS text messages plus treatment as usual for the management of patients with MDD were 0.85 and 0.67, using a 2-sided significance level (α) of .05, and a power of 80% (β=.2). Thus, in this pilot trial, we anticipate that a similar sample size of 80 patients with MDD, with 40 patients allocated into each study arm, will be adequate to detect differences in outcome measures between the 2 interventions.

### Randomization

The study used a computer-generated block randomization to ensure balance between the 2 intervention groups. Randomization codes were provided via SMS text message directly to the blinded researcher’s password-protected phone line with a secure online backup. This occurred during enrollment as each participant signed the consent form. Participants were asked to use either the email address or phone number with which they received messages as their study ID on the follow-up online surveys.

### Interventions

Commencing 1 day after enrollment, participants received daily supportive messages, either through SMS text message or email. However, the study did not specify the device upon which the email group should receive the emails. The contents of both types of messages were the same and were designed by mental health professionals and mental health service users, based on the principles of CBT, including addressing automatic thoughts, cognitive distortions, and the underlying beliefs that perpetuate depressive symptoms. The delivery time for each message was set for 10 AM Mounty Time and participants received these messages for 6 months regardless of whether they were delivered via SMS text message or email. This duration corresponds to a minimum of 180 days of message delivery. The supportive messages maintained consistent length and structure across both delivery methods (SMS text message and email). A sample of the supportive messages include, (1) if you feel overwhelmed or unable to cope, reach out for help. Talk to your friends, family, and mental health team. We all need help at some point in our lives. We all need help at some point in our lives!—Michael Holley; (2) we can change the way we feel by changing the way we think and behave. The first step to change is knowing yourself better. For a couple of days, journal your thoughts, emotions, and behaviors; and (3) depression zaps our energy and our desire to do things. The problem is we stop doing things that make us feel better, so we feel worse. Make an effort to do things that you enjoy, such as watching a movie with family or going for a walk.

While individual preferences for supportive messages may vary, these CBT-based supportive SMS text messages have been previously successfully used in 2 randomized controlled trials involving individuals with MDD and were selected to address common challenges and encourage coping strategies relevant to individuals experiencing depression or similar mental health concerns [31,32].

### Data Collection

After the signing of the informed consent, participants were randomized into 1 of the 2 groups (email message or SMS text message) and asked to complete a baseline assessment questionnaire encompassing both demographic and clinical data. This was done in person. Further, at 6 months, an online survey link along with an invitation to complete the assessment was sent to all participants either via email or SMS text messages. Data were collected between January and July 2021. Also, there was no compensation plan in any form included in our protocol.

### Outcome Measures

To assess the effectiveness of the 2 interventions under study, the mean scores of the outcome measures were compared on specified scales at baseline and at 6 months for both study arms. The depression symptom scores were generated from the 9-item PHQ-9 scale [41]. This validated instrument with a Cronbach α of 0.89 is mostly used for diagnosing and gauging the severity of depression in general medical and mental health care settings. Each of the 9 questionnaire items is graded on a scale of 0 (not at all) to 3 (nearly every day). Higher scores on the scale indicate an elevated level of depression. The PHQ-9 has demonstrated good convergent validity with related constructs and possesses adequate internal consistency [42].

For symptoms of anxiety, the GAD-7 scale [43] was used to evaluate the levels of anxiety at baseline and 6 months for each participant in both study arms. The 7-item validated GAD-7 scale with a Cronbach α of 0.92 gauges self-reported anxiety levels in respondents, with each item on the scale scored between 0 (not at all) to 3 (nearly every day). Higher scores on the scale signify elevated levels of anxiety. The quality of life of participants was evaluated using the WHO-5 [44]. This brief 5-item questionnaire assesses the subjective well-being of respondents and has proven validity as an outcome measure in clinical trials and as a generic scale for tracking well-being over time or between groups. The WHO-5 was good (Cronbach α=0.858) [45,46]. The use of these 3 scales (PHQ-9, GAD-7, and WHO-5) stems from their established validity, reliability, and widespread use in clinical and research settings of the study area.

### Statistical Analyses

Data were analyzed using SPSS (version 26; IBM Corp) for Windows [47] and were reported following the CONSORT (Consolidated Standards of Reporting Trials) guidelines (Multimedia Appendix 1) [48]. Descriptive data for baseline parameters were presented using frequencies and percentages among the 2 intervention groups and compared by chi-square for categorical variables.

To compare the effectiveness of the 2 interventions in the management of depressive symptoms, a 1-way between-groups analysis of covariance (ANCOVA) was used to compare the changes in mean score from baseline to 6 months on all the measuring scales used while controlling for baseline scores. Before conducting the ANCOVA test, preliminary checks were performed to ensure no violation of assumptions of normality, linearity, homogeneity of variance, homogeneity of regression slopes, or reliable measurement of the covariate. The intervention group served as the independent variable, while the dependent variable consisted of the 6-month score on the various measures (PHQ-9, GAD-7, and WHO-5), and the covariates consisted of the baseline scores on the measures. The significance criterion for statistical tests was set at a 2-tailed α-level of *P≤*.05.

### Ethical Considerations

All study participants were provided with an information leaflet and offered the opportunity to ask questions about the study before being asked to provide written informed consent to participate in the study. Data collection occurred online through patient self-completed rating scales. Participants were equipped with resources for support in case of mental or psychological distress. Specifically, we included contact details for the Alberta Health Services emergency helpline, along with information about the principal investigator, in the patient information leaflet, the consent form, and as part of the introductory SMS text message or email message to patients. The study received institutional review board approval from the University of Alberta Human Ethics Review Board (Pro00105429), and the trial was registered at ClinicalTrials.gov (NCT04638231).

## Results

**Figure 1:**
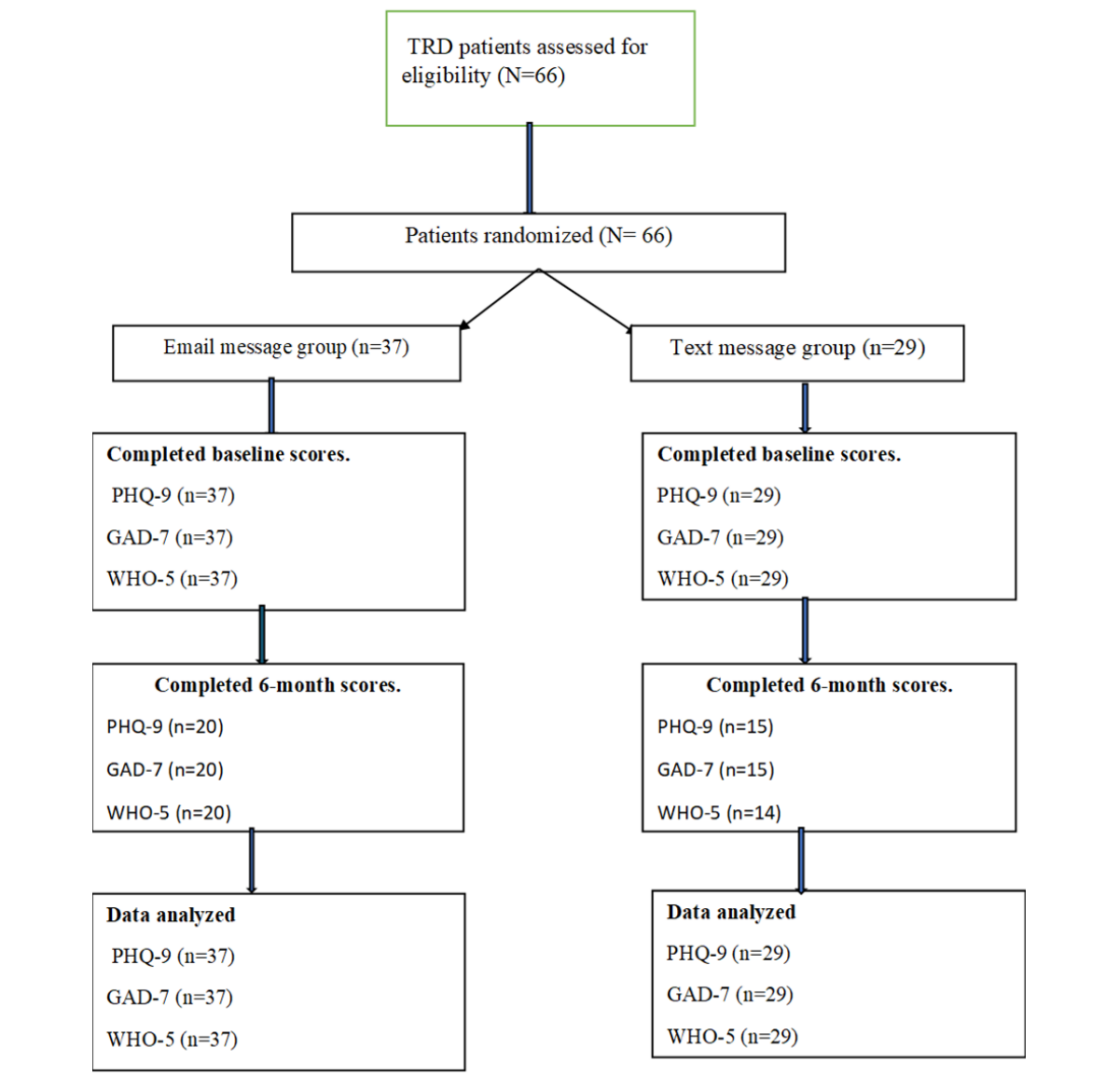
Study flowchart. GAD-7: Generalized Anxiety Disorder-7; PHQ-9: Patient Health Questionnaire-9; TRD: Treatment Resistant Depression; WHO-5: World Health Organization Well-Being Index.

The study realized a total of 66 participants who were randomized into the 2 intervention groups. Thus, 37 participants were enrolled in the email messages group, while 29 participants were placed in the SMS text messages group. Figure 1 is the study flowchart.

Table 1 provides the baseline distribution of sociodemographic and clinical characteristics of the 2 study groups. Most of the participants were females (n=43, 65%), aged between 26 and 40 years (n=34, 55%), had high school education (n=35, 58%), employed (n=33, 50%), single (n=24, 36%), and belonged to the Caucasian ethnic group (n=49, 74%). Except for age (*P*=.01), the chi-square analysis revealed no statistically significant differences in baseline sociodemographic characteristics between the 2 intervention groups (email messages and SMS text messages; *P*>.05).

Regarding clinical variables, 56 (85%) had no history of any major physical illness and 61 (92%) responded “No” to having a history of admission for treatment of mood disorders. With medication, respondents having a history of being on antidepressants were 30 (46%), a history of mood stabilizers was 1 (2%), a history of antipsychotics was 1 (2%), a history of nocturnal sedation was 1 (2%), and history of tranquilizers was 1 (2%). Like the sociodemographic variables, the chi-square analysis revealed no statistically significant differences in baseline clinical characteristics between the 2 intervention groups (email messages and SMS text messages) indicated by the respective *P* values (*P*>.05).

As displayed in Table 2, a 1-way between-group ANCOVA was performed to compare the effectiveness of the 2 interventions (email and SMS text messages). After adjusting for the baseline scores on the PHQ-9, there was no significant difference in the means between the 2 intervention groups at 6 months. After imputation of baseline data for missing 6-month data, there remains no significant difference in the mean changes in PHQ-9 scores from baseline to 6 months between the email messages and SMS text messages groups (*P*>.05). Overall, only 2.1% of the variance in the dependent variable (6 months PHQ-9 scores) was explained by the independent variable.

On the GAD-7 scale after controlling for the baseline scores, there were no significant differences between the 2 intervention groups at 6 months. After data imputation, there was no significant difference in the mean changes in GAD-7 scores from baseline to 6 months between the email message and SMS text message groups (*P*>.05). There was a relationship between the baseline intervention score and the 6-month intervention score on the GAD-7 scale, as evidenced by the partial η^2^ value of 0.09. The intervention type explained only 2.6% of the variance in the dependent variable (6-month mean GAD-7 scores).

For the World Health Organization (WHO), after adjusting for baseline scores, there were no significant differences between the groups at 6 months, as shown in Table 2. Furthermore, only 1.1% of the variance in the dependent variable (6 months mean WHO-5 score) was explained by the independent variable.

**Table 1:** Distribution of baseline demographic and clinical characteristics between the 2 study groups.

Variable		Email, n (%)	SMS text message, n (%)	Total, n (%)	Chi-square (*df*)		*P* value	
**Sex**						2.6 (1)		.11
	Male	16 (43)	7 (24)	23 (35)				
	Female	21 (57)	22 (76)	43 (65)				
**Age (years)**						10.2 (2)		.01
	≤25	12 (36)	1 (3)	13 (21)				
	26-40	14 (42)	20 (69)	34 (55)				
	≥41	7 (21)	8 (28)	15 (24)				
**Formal educational level**						0.7 (2)		.70
	Elementary	1 (3)	2 (7)	3 (5)				
	High school	19 (58)	16 (59)	35 (58)				
	College or university	13 (39)	9 (33)	22 (37)				
**Employment status**						4.8 (3)		.19
	Employed	17 (46)	16 (55)	33 (50)				
	Unemployed	14 (38)	11 (38)	25 (38)				
	Retired	1 (3)	2 (7)	3 (5)				
	Student	5 (14)	0 (0)	5 (8)				
**Marital status**						5.1 (4)		.28
	Single	15 (41)	9 (31)	24 (36)				
	Partnered	9 (24)	3 (10)	12 (18)				
	Cohabiting	4 (11)	6 (21)	10 (15)				
	Married	3 (8)	6 (21)	9 (14)				
	Divorced or separated	6 (16)	5 (17)	11 (17)				
**Ethnicity**						0.2 (4)		.99
	Caucasian	28 (76)	21 (72)	49 (74)				
	Indigenous	3 (8)	2 (7)	5 (8)				
	Asian	4 (11)	4 (14)	8 (12)				
	Black	1 (3)	1 (3)	2 (3)				
	Other	1 (3)	1 (3)	2 (3)				
**History of major physical illness**						0.2 (1)		.68
	Yes	5 (14)	5 (18)	10 (15)				
	No	32 (87)	24 (83)	56 (85)				
**History of admission for treatment of mood disorders**						0.6 (1)		.45
	Yes	2 (5)	3 (10)	5 (8)				
	No	35 (95)	26 (90)	61 (92)				
**History of antidepressants**						0.8 (1)		.37
	Yes	15 (41)	15 (52)	30 (46)				
	No	22 (60)	14 (48)	36 (55)				
**History of mood stabilizers**						0.8 (1)		.37
	Yes	1 (3)	0 (0)	1 (2)				
	No	36 (97)	29 (100)	65 (99)				
**History of antipsychotics**						0.8 (1)		.37
	Yes	1 (3)	0 (0)	1 (2)				
	No	36 (97)	29 (100)	65 (99)				
**History of nocturnal sedation**						1.3 (1)		.26
	Yes	0 (0)	1 (3)	1 (2)				
	No	37 (100)	28 (97)	65 (99)				
**History of tranquilizers**						0.8 (1)		.37
	Yes	1 (3)	0 (0)	1 (2)				
	No	36 (97)	29 (100)	65 (99)				

**Table 2:** Descriptive mean scores of outcome measures and ANCOVA^a^ test parameters for the email message and SMS text message groups.

Measure	Baseline, mean (SD)		Discharge, mean (SD)		Mean difference (95% CI)	*F* test (*df*)	*P* value	Partial eta
	Email	SMS text message	Email	SMS text message				
PHQ-9^b^	19.65 (5.37)	19.76 (5.05)	12.05 (8.97)	15.47 (5.55)	–1.90 (–6.53 to 2.74)	0.70 (1, 32)	.41	0.02
GAD-7^c^	14.70 (5.55)	15.66 (4.59)	18.75 (14.24)	15.33 (9.70)	5.78 (–1.94 to 13.50)	2.33 (1, 32)	.14	0.07
WHO-5^d^	15.24 (14.48)	15.85 (13.02)	31.80 (25.87)	19.71 (17.85)	11.85 (–3.81 to 27.51)	2.38 (1, 31)	.13	0.07

^a^ANCOVA: analysis of covariance.

^b^PHQ-9: Patient Health Questionnaire-9.

^c^GAD-7: Generalized Anxiety Disorder-7.

^d^WHO-5: World Health Organization Well-Being Index.

## Discussion

### Principal Findings

This noninferiority pilot trial involved 66 participants who were randomized to receive either CBT-based daily supportive email or SMS text messages for 6 months. Descriptive data indicated no significant differences in sociodemographic and clinical characteristics between the 2 groups under study. The analysis followed an intention-to-treat approach and the result revealed none of the 2 modalities was inferior to the other, as demonstrated by the no significant differences in the mean scores of depression, anxiety levels, and well-being scales between the 2 groups. The positive findings related to depression and well-being symptom improvement, although not conclusive, offer insights into the effectiveness of these innovative e-mental health interventions in addressing depression symptoms and improving well-being. Our result is consistent with a previous study in which the Kokoro app (Apple Inc), a smartphone‑based CBT program, was found to show feasibility and acceptability as an add‑on therapy for treatment‑resistant depression [49]. Furthermore, in a related systematic review, the findings suggested that mobile phone–based technology could be used to deliver psychotherapeutic interventions across a range of mental health disorders. These positive findings may be because mobile phone apps allow patients and health care providers to track symptoms and early changes collaboratively; hence, facilitating a need‑based allocation of resources promptly [50].

Interestingly, anxiety symptoms did not improve with the intervention and worsened in the group that received the messages via email, although the change in mean anxiety symptom scores was not statistically different between the 2 groups. The fact that patients recruited into this study have a primary MDD diagnosis and the CBT-based messages were designed to address anxiety symptoms may explain the lack of positive impact of the messages on anxiety symptoms in comparison to depression and well-being symptoms as seen in results of the population level Text4Hope program, which produced the highest reductions in anxiety symptoms [51-53]. The impact of the program messages on depression symptom using both modalities align with previous studies, indicating a significant reduction in depressive symptoms among participants [30,31].

The effectiveness of email as a communication tool can be examined through the framework of media richness theory [54], which suggests that a medium’s ability to convey information depends on whether it is used during periods of uncertainty thus, when there is insufficient information or when there is confusion or ambiguity. Email is positioned on the less rich end of the spectrum primarily due to its limited provision of immediate feedback and multiple cues. However, email can effectively address uncertainty by accommodating a large volume of information [55]. The exploration of email as an alternative delivery method for supportive messages for individuals with mental health concerns is timely. Emails offer unique advantages, such as longer message lengths and asynchronous communication. While emails have established themselves as secure channels for health communication, their efficacy in delivering mental health interventions remains underexplored. This study’s findings come in handy in helping to bridge this knowledge gap, albeit with a smaller-than-anticipated sample size. Though our study findings did not display any clear superiority of 1 modality over the other as demonstrated on all measures, both intervention groups (email and SMS text messaging) demonstrated potential in reducing depressive symptoms and enhancing the quality of life of the participants. This lack of a significant difference between the interventions suggests that both modalities could be viable options for mental health care delivery, depending on individual preferences and circumstances.

Delving into the clinical relevance of the findings is crucial for understanding their implications in real-world settings. While the results may indicate no statistically significant differences between interventions, it is essential to highlight the practical significance for both patients and health care providers. For patients, even subtle differences in platforms or interventions could have meaningful implications for their experience and outcomes. Factors such as ease of access, user interface, and the overall patient-provider interaction can significantly impact patient satisfaction, engagement with treatment, and ultimately, treatment outcomes. Therefore, even if statistically noninferior, identifying any trends or patterns in patient preferences or experiences across different platforms can inform the optimization of interventions to better meet patient needs and enhance overall care quality.

From the perspective of health care providers, understanding the practical significance of differences between interventions can inform decision-making regarding resource allocation, intervention implementation, and patient management strategies. For example, identifying specific features or aspects of interventions that contribute to patient satisfaction or engagement can guide the selection and customization of interventions to align with patient preferences and optimize treatment delivery. Moreover, considering the broader context of health care delivery, such as resource constraints, technological infrastructure, and health care disparities, can further illuminate the practical implications of the findings. For instance, interventions that are more accessible, cost-effective, and scalable may hold greater relevance and potential for widespread adoption, particularly in settings with limited resources or underserved populations.

Therefore, while the absence of statistical differences between interventions is noteworthy, evaluating their clinical relevance provides valuable insights into their potential impact on patient care, treatment outcomes, and health care delivery practices. By considering the practical significance of findings, we can better inform decision-making, enhance patient-centered care, and ultimately improve health outcomes in real-world settings.

By interpreting the study findings against the existing literature, it is essential to acknowledge that this study used a noninferiority trial design. This design aimed to demonstrate that the new intervention (email messaging) is not worse compared to the standard intervention (SMS text messaging) in delivering daily supportive messages for the management of MDD. Therefore, the focus is on equivalence rather than superiority. Hence, it is imperative to consider the context of comparative efficacy when translating the findings.

The study findings aligned with those of a randomized comparative investigation titled “Web- and Mobile App–Based Mental Health Promotion Intervention Comparing Email, Short Message Service, and Videoconferencing Support for a Healthy Cohort.” In this study, significant improvements were noted within each group from pre- to postintervention across all outcome measures (*P*≤.001). However, there were no significant differences between groups for any outcome measure, including mental health (*P*=.77), vitality (*P*=.65), depression (*P*=.93), anxiety (*P*=.25), and stress (*P*=.57). This indicates that SMS text messages did not yield significant advantages over automated email support [56]. However, conversely, to our findings and in a related study on a mobile phone–based service preferences among 75 patients, with 49.3% diagnosed with bipolar disorder and 50.7% with schizophrenia, the majority preferred SMS text messages (n=14, 19%) over email (n=1, 1%) as their service delivery medium [57]. Additionally, a systematic review and meta-analysis examining web-based interventions using supplementary support methods indicated that SMS exhibited substantial effects (*d*=0.81) compared to email (*d*=0.18) [58]. This preference may stem from the ease and convenience of SMS text messaging, especially during symptom fluctuation or cognitive challenges associated with bipolar disorder and schizophrenia. The study findings contribute to a deeper understanding of the transformative potential of digital mental health care platforms in addressing the global burden of MDD. Aligning the findings with standard knowledge emphasizes the relevance and potential therapeutic impact of incorporating technology in mental health care delivery. The study’s recognition of the extensive accessibility of mobile networks globally is consistent with literature affirming the rapid rise in internet accessibility and mobile phone usage [19-21]. The integration of mHealth in the delivery of mental health interventions is consistent with the increasing reliance on digital platforms for health care.

The finding that SMS text messages and emails may have comparable effectiveness in mitigating depression symptoms in patients with MDD could be attributed to several factors. While both mediums offer a means of delivering supportive content, they differ in their immediacy, format, and perceived level of personalization. SMS text messages are often perceived as more immediate and personal due to their shorter length and direct delivery to individuals’ phones. On the other hand, emails may offer a more formal and comprehensive platform for conveying information, potentially allowing for more detailed messages and attachments. Additionally, individual preferences and access to technology may play a role in how patients engage with and respond to messages delivered via SMS text message or email. Further research exploring these differences in message delivery and their impact on patient outcomes could provide valuable insights into optimizing digital interventions for mental health support.

### Study Implications

The study’s findings contribute to the growing body of literature on digital mental health interventions. While both email and SMS text messaging interventions demonstrated comparable outcomes, the distinctive circumstances of participants lacking active personal cell phones and telephone numbers highlight the significance of considering alternative means of health care service delivery. The study’s aim of exploring novel psychotherapeutic interventions that are cost-effective, scalable, and accessible emphasizes the growing call in the literature for embracing digital solutions in mental health care practices. Understanding subtle differences in interventions is crucial for both patients and health care providers as it impacts patient satisfaction, engagement, and treatment outcomes, informing decision-making for resource allocation, intervention implementation, and patient management strategies, especially when considering broader health care contexts such as accessibility and cost-effectiveness. With these patients who were disadvantaged by having no cell phones could still be reached and supported via emails. Patients have the option to choose between the 2 approaches, as they are equally effective. Also, not all patients will have either a cell phone or a functional and accessible email account, but most patients will have at least 1 of these communication modalities which means the supportive messages can reach more patients. Future studies should build on these findings to refine and expand the scope and use of digital interventions for the management of individuals diagnosed with depressive conditions. The study addresses a critical gap in the literature by exploring the effectiveness of email messaging as a secure digital mode for mental health communication. Thus, depending on an individual’s preferences, needs, and technological access, mental health service providers can offer a range of digital solutions, from email to app-based platforms, ensuring a more personalized approach to care.

### Limitations and Future Directions

The smaller-than-expected sample size may have impacted the statistical power of our analyses. Therefore, the results of the study should be interpreted with caution, and a further study with a larger sample size is needed to validate the findings. Another important limitation is that the study used self-reported instruments as opposed to a formal diagnostic interview to evaluate clinical variables. It is important to note that while these instruments are validated scales, they do not serve as diagnostic tools. Furthermore, the study’s noninferiority design assumes that both interventions (email and SMS text messaging) are equally effective as an augmentation treatment for individuals with depression concerns, which may not account for potential differences in user experience or engagement. Again, the study did not specify the electronic devices used for receiving the email messages and, hence, did not control the devices. This intervention was designed as supplementary support for individuals with MDD rather than a stand-alone treatment. We did not assess the different types of therapies patients were receiving. Thus, it is possible that the overall improvement in mood symptoms reported in both groups is not attributable to the supportive SMS text messages or email messages. However, this research supplements previous randomized controlled studies which involved controlled patient populations [31,32] and other naturalistic controlled population-level studies [59,60] which have established the effectiveness of daily supportive SMS text message interventions as an adjunctive therapy for treating MDD.

Future directions from the study include the need for larger sample sizes to validate findings, particularly in confirming email messaging’s effectiveness compared to SMS text messaging for mental health intervention. Further exploration into user experience and engagement differences between these platforms is needed, alongside implementing controls for device use. Additionally, future research could explore the synergistic effects of the daily supportive messages either via SMS text message or email in combination with different therapeutic approaches to optimize treatment outcomes for individuals with MDD.

### Conclusions

This randomized pilot trial provides preliminary insights into the pot of email messaging as an alternative delivery mode for supportive content to patients with MDD. The findings did not reveal evidence of the inferiority of email messaging compared to the more established SMS text messaging approach over the 6-month study period. While email and SMS text message groups demonstrated comparable outcomes on depression, anxiety, and well-being measures, the small sample size limits definitive conclusions regarding their therapeutic equivalence. These early-phase results lend cautious support to the premise that widely accessible platforms such as emails could expand options for delivering supportive mental health content.

## Appendix

Multimedia Appendix 1 CONSORT-eHEALTH (V 1.6.1).

## Abbreviations

ANCOVA: analysis of covariance
BDI: Becks Depression Inventory
CBT: cognitive behavioral therapy
CONSORT: Consolidated Standards of Reporting Trials
DSM-5: Diagnostic and Statistical Manual for Mental Disorders (Fifth Edition)
GAD-7: Generalized Anxiety Disorder-7
MDD: major depressive disorder
mHealth: mobile health
PHQ-9: Patient Health Questionnaire-9
WHO-5: World Health Organization Well-Being Index

## Data Availability

The data sets generated and analyzed during this study are available from the corresponding author on reasonable request.
